# Temporal artery biopsy in giant cell arteritis: clinical perspectives and histological patterns

**DOI:** 10.3389/fmed.2024.1453462

**Published:** 2024-09-25

**Authors:** Pavlos Stamatis, Carl Turesson, Aladdin J. Mohammad

**Affiliations:** ^1^Rheumatology, Department of Clinical Sciences, Lund University, Lund, Sweden; ^2^Rheumatology, Sunderby Hospital, Luleå, Sweden; ^3^Rheumatology, Department of Clinical Sciences, Lund University, Malmö, Sweden; ^4^Department of Medicine, University of Cambridge, Cambridge, United Kingdom

**Keywords:** giant cell (temporal) arteritis, temporal artery biopsy, histology, specimen length, arteritis, adventitial inflammation, transmural inflammation, polymyalgia rheumatica (PMR)

## Abstract

Although its role has been debated, temporal artery biopsy (TAB) remains the gold standard for the diagnosis of cranial giant cell arteritis (GCA). The specificity of TAB is excellent and the sensitivity, albeit lower, is comparable with other diagnostic modalities used for the diagnosis of GCA. This outpatient procedure has a low rate of complications and is well integrated in the majority of healthcare systems. The length of the specimen, the number of the examined sections and the prolonged use of glucocorticoids before the biopsy may affect the outcome of the TAB as diagnostic tool. The typical histological findings in GCA are often characterized by granulomatous inflammation with infiltration of mononuclear cells with or without the presence of giant cell, varying degrees of external and internal elastic lamina damage and intimal thickening. Overlooking signs of inflammation in the adventitia and in connective tissue surrounding the temporal artery may lead to false negative results. The distinction between healed arteritis and age-related atherosclerosis may be challenging.

## Introduction

Since 1932, when Bayard Horton reported the outcomes of the first two temporal artery biopsies (TABs) of patients with giant cell arteritis (GCA) and until the very recent past, TAB was the only diagnostic procedure which could confirm the diagnosis of GCA (1, 2). During the last two decades, the increased recognition of the extracranial features of the disease and the use of imaging studies, including ultrasound, for the diagnosis of both cranial and extracranial GCA have challenged the role of TAB for the diagnosis of GCA. Currently, there is a discrepancy between the recommendations of the European Alliance of Associations of Rheumatology (EULAR) and the recommendations of the American College of Rheumatology (ACR) for the diagnostic role of TAB. EULAR recommends imaging, particularly temporal and axillary ultrasound, as first diagnostic modality to investigate mural inflammatory changes (3). On the other hand, ACR recommends TAB over temporal artery ultrasound (4). Differences in the technical expertise of healthcare professionals between different healthcare systems and the severe consequences of missing the diagnosis (visual complications, stroke) as well as the burden of the side effects due to unnecessary long-term treatment with glucocorticoids (GCs) in cases of false positive findings may explain this discrepancy. A recent Cochrane meta-analysis could not draw any conclusions on whether the halo sign on temporal artery can replace TAB for diagnosing GCA as data were heterogeneous and the included studies did not use the same halo thickness threshold or did not report it (5).

It has been reported that in areas with high availability of trained ultrasonographers, the proportion of GCA patients diagnosed using TAB has decreased in recent years (6). If TABs are reserved for atypical cases, this might lead to a reduction in the sensitivity of GCA. On the other hand, if patients are selected for TAB based on clinical expertise and ultrasound findings suggesting vasculitis, this would increase sensitivity.

The aims of this narrative review are (1) to provide an update on some important clinical parameters regarding TAB in GCA (such as rate of complications, unilateral vs. bilateral biopsy, specimen length, number of examined sections, predictors of positive TAB and effect of therapy on the specimen) and (2) to describe the histological patterns seen in GCA in order to assist clinicians in the interpretation of TAB findings, with optimization of the TAB use in every day clinical praxis as the ultimate goal.

## Methods of literature search

We conducted a PubMed search on May 12, 2024, for English-language articles, using the following keywords: giant cell arteritis, biopsy, histolog* and temporal artery. Reference lists of retrieved articles were also manually reviewed to identify additional relevant studies. The initial search revealed 114 studies. A careful review of the most relevant studies (*n* = 23) formed the basis for this narrative review.

## Clinical perspectives

TAB has been considered the gold standard for diagnosis of GCA, especially for the cranial phenotype. A meta-analysis comprising 32 studies conducted from 1993 to 2015 reported TAB sensitivity of 77% for GCA (7). Two studies after this meta-analysis have reported significantly lower sensitivity 33% (95%CI; 19–51%) and 39% (95%CI; 33–46%) (8, 9). It is possible that patient selection (ophthalmology center, low proportion with headache) (8) and lack of structured training of surgeons and pathologists (9) may partly explain these results. The specificity of TAB is excellent and up to 100% in several studies (9–13). The likelihood of a positive biopsy increases with better selection of patients with high probability of cranial GCA. We reviewed reports on more than 6,500 TABs performed between 1997 and 2019 in southern Sweden and found that only 21% were positive for GCA (14). The proportion of positive TABs in our study should be interpreted cautiously as it was originated from an unselected population (all patients in our region who underwent TAB for any reason between 1997 and 2019). However, in studies of patients in whom diagnosis was confirmed by clinical and laboratory characteristics and who fulfilled the classification criteria for GCA (15), proportions with positive TAB have been reported to be 77% (16) and 87% (17), respectively, in two studies of populations with a high incidence of GCA from Malmö, Sweden and Minnesota, USA (16, 17).

In the TABUL study (The Role of Ultrasound Compared to Biopsy of Temporal Arteries in the Diagnosis and Treatment of Giant Cell Arteritis), 14 pathologists evaluated 30 TABs from patients with suspected GCA (9). In 11 cases the pathologists agreed unanimously, in 13 cases there were only one or two pathologists with different opinion from the majority but in 6 cases the opinion was divided. The results from the TABUL study imply that despite the long-established use of TAB for the diagnosis of GCA, there still exist areas for continued improvement. A better selection of patients undergoing TAB, an adequate specimen length and a sufficient number of examined sections are modifiable factors which may increase the diagnostic accuracy of TAB. Standardization of terminology and a consensus among healthcare professionals who are involved in the management of patients with GCA on processing, interpretation and reporting of TAB specimens are also key components of the real-world application of TAB in every day clinical praxis (18, 19).

The involvement of a specialist team in investigation of suspected GCA, as recommended by EULAR (20), likely reduces the number of TABs performed with a low pre-test probability of GCA.

### Procedural aspects

A TAB is usually performed under local anaesthesia as an outpatient procedure. It is recommended that it should be performed by an experienced surgeon. The rate of complications is low in such cases (0.5%), with the most serious complications being facial palsy and scalp necrosis (21–25). In some cases, sampling errors may arise when a vein or other anatomical structure is sampled instead of an artery (9).

### Clinical features predicting of positive biopsy

Reported weight loss at baseline, age ≥ 75 years, female sex, headache, jaw claudication, neck pain, elevated ESR, and elevated platelet levels have been reported as predictors of a positive TAB in patients with suspected GCA (26–29). A study of 459 positive for GCA TABs (out of 3,001 individuals who underwent TAB) found that the odds of a positive TAB were 1.5 times greater with an ESR ranging from 47 to 107 mm/h, 5.3 times greater with CRP > 2.45 mg/dL and 4.2 times greater with platelets >400.000/μL (30). Among patients with a negative biopsy, fulfillment of the ACR criteria, PMR and high platelet count have been reported to be the best predictors for GCA diagnosis (31).

### Unilateral versus bilateral TAB

Several observational studies have shown that a bilateral TAB increases diagnostic accuracy by 3–14% (25, 32–37). A large retrospective study over all three Mayo Clinic campuses included 3,817 TABs. Of the 603 patients with bilateral biopsy within 3 months from the initial biopsy, 43 (7%) had a negative initial biopsy followed by a positive on the other side (38). Although this indicates some improvement in the diagnostic yield, it seems to be moderate, and therefore a bilateral temporal artery is recommended only for selected cases with discordance between the clinical findings and the findings described in the initial TAB report.

### Specimen length

Skip lesions are reported to occur in 8.5–28% of TAB+ GCA (39, 40). The optimal length of a TAB to minimize the risk of a false negative result is a matter of debate. EULAR recommends a specimen of at least 10 mm in length, which corresponds to a post-fixation length of at least 7 mm (20). A recent study from Sweden showed that the temporal artery contracted by about 12% after surgical excision, both in positive and negative TABs, while formalin fixation caused no further shrinkage (41). Another study from the US, found a 20% mean percentage of contraction, 12% (SD 7%, range 0–25%) in TAB+ specimens and 22% (SD 9%, 7–45%) in TAB- specimens (42). Taken altogether, and based on several observational studies, a post-fixation length of 5–10 mm, which corresponds to a prefixation surgical specimen length of 10–15 mm, is considered sufficient for diagnosis (27, 32, 43, 44).

### Multiple sectioning

After the TAB, the extracted temporal artery specimens are transversally sectioned into smaller pieces (measured in mm), fixed with formalin and completely embedded in paraffin (usually transversally) (32, 45). Then, sections measured in μm are cut from paraffin blocks and stained most commonly with hematoxylin–eosin (32, 45). As the first section could be negative, at least three sections at deeper levels should be examined (32, 44). Muratore et al. reviewed 662 TABs performed for suspected GCA with 65% of the specimens classified as negative (44). The authors found that 26 out of 408 TAB specimens (6.4%) reported initially as uninflamed, had inflamed sections, after cuts of additional biopsy sections at deeper levels (44). In 14/26 specimens the inflamed section was the second, in 9/26 specimens the inflamed section was the third and in 3/26 specimens the inflamed section was the fourth (44). Examination of multiple sections at deeper levels is of importance especially in cases of inflammation restricted to periadventitial and/or adventitial tissue (32, 44).

### Impact of treatment on histological findings

Existing evidence suggests that the inflammatory findings in TAB subside more slowly than do imaging findings. When the temporal artery is affected, histological evidence of ongoing inflammation is present in the TAB for at least a month after therapy initiation (16, 46), and positive histological findings have been reported up to 12 months after GCA diagnosis, especially when symptoms are present (45, 47, 48). In a study with repeated TABs, 44% of patients with initial positive biopsies also had positive biopsies when having symptoms of active disease between 9 and 12 months after therapy initiation (45). In our clinical practice, we aim to obtain a TAB within 2 weeks of treatment initiation to confirm or rule out the diagnosis and to avoid unnecessary medication toxicity in patients with negative TAB and low clinical suspicion for GCA. This timeframe is also recommended, with low level of evidence, in the 2021 ACR/Vasculitis Foundation guidelines for the management of GCA (4). On the other hand, when a TAB has not been previously conducted, and there is high clinical suspicion of GCA with typical symptoms present, the results of a TAB could be informative, even if the patient has received GC treatment for more than 2 weeks (16, 45, 46). Low GC doses in GCA patients with prior PMR seem to not affect histological findings of vasculitis (46).

## Histological patterns in GCA

### The typical pattern

Granulomatous inflammatory infiltrate comprised mainly of CD4+ lymphocytes and macrophages, usually affecting all three artery layers, is considered to be the hallmark of a positive TAB. Transmural inflammation is the most common pathological pattern in inflamed temporal arteries of patients with GCA. In artery cross-section, the inflammatory infiltrate appears as concentric rings, with a thicker inflammatory ring adjacent to external elastic lamina and a thinner ring along the internal elastic lamina (32). Mono- and multi-nucleated giant cells are present along the internal elastic lamina in the majority of positive TABs (32, 45, 46), but the absence of giant cells does not preclude a GCA diagnosis. In a study of patients with evidence of inflammation indicating GCA in TAB, absence of reported giant cells was associated with involvement of the aorta and its branches, suggesting that cranial arteritis with typical TAB findings and large vessel involvement are different parts of the spectrum of GCA (49).

Although inflammation affecting the media is traditionally considered a defining feature of a positive TAB, the media is relatively spared except for severe cases in which the inflammatory infiltrate is diffuse, severely affecting all three arterial layers (panarteritis). The intima becomes progressively thicker due to myofibroblastic proliferation, resulting in varying degrees of occlusion of the lumen. The grade of intimal thickening correlates with the severity and intensity of the inflammatory infiltration (46). Occlusion due to thrombosis occurs rarely (32). Fragmentation of internal elastic lamina and neo-angiogenesis are commonly seen in positive TABs (32, 50). Table 1 summarizes reported histological findings in patients with TAB+ GCA from 5 studies (32, 45, 46, 50, 51).

**Table 1:** Histological findings in patients with positive TAB with transmural inflammation or panarteritis.

	Cavazza et al. (32)	Hernandez-Rodriguez et al. (46)	Maleszewski et al. (45)	Putman et al. (50)	Font and Prabhakaran (51)
Number of + Biopsies	354	285	40	705	35
Cell types					
Lymphocytes	100%	NR	100%	NR	100%
Plasma cells	Inconspicuous	NR	83%	NR	NR
Giant cells	74.8%	61.4%	55%	51%	42.9%
Eosinophils (%)	8%	NR	18%	NR	NR
Neutrophils (%)	1.8%	NR	3%	NR	NR
Isolated adventitial or periadventitial inflammation	22.5%	5.6%	NR		22.9%
Disruption of internal elastic membrane	Very common	NR	100%	41%	100
Intimal thickening	100%^a^	72.3%	93%	33%	NR
Thrombus	9.5%	NR	NR	4%	NR

a In patients with transmural inflammation (*n* = 274).

NR, not reported. Data derived from quantitative studies.

Investigation of cellular markers or cytokines is not currently part of standard evaluation of TABs. Detailed studies have revealed expansions of T cell subsets, with reduction of Th17 but not Th1 pathways after GC therapy (52). Such findings may have implications for future targeted therapies and possibly also for disease monitoring.

### Periadventitial and adventitial inflammation

Temporal artery biopsies with mild inflammatory lesions and biopsies from patients with early GCA may lack the described typical features. The inflammation occurs as a dynamic process in which the inflammatory infiltrate spreads through the wall of the temporal artery from the adventitia toward the intima (46). Consequently, at the time of temporal artery excision, inflammation may be restricted to the periadventitial or adventitial tissue, as the small vessels around the temporal artery and the adventitial vasa vasorum are considered the gates through which the invading inflammatory cells initiate the inflammatory process, as well as the primary field in which it takes place. A series of 354 TABs showing inflammation included 80 (22.5%) with inflammatory cell infiltrates restricted to the adventitia or the periadventitial tissue (32). Such isolated inflammation may escape the pathologist’s attention (9, 32, 53). As mentioned, the first section of a specimen may occasionally be negative, and examination of deeper sections may be necessary to detect inflammation, especially when it is limited to adventitia and the surrounding connective tissue (32, 53). A negative first section appears to be infrequent in TABs of patients with transmural inflammation, whereas it occurs in 32–50% of biopsies of patients with isolated periadventitial or adventitial inflammation (32). Table 2 presents the most frequent histological patterns in inflamed TAB and some of its clinical significance (32, 45, 46, 50, 51, 53–57).

**Table 2:** The most frequent histological patterns seen in TABs of patients with biopsy-confirmed GCA based on selected studies.

Pattern	Description	Clinical significance
Normal artery	The three arterial layers are separated by the external elastic lamina (adventitia-media) and internal elastic lamina (media-intima). The vasa vasorum is located in the adventitia. At the outer limit of the adventia, connective tissue consisting of adipose tissue contains small vessels with or without a muscle layer. Small nerves may exist in the periadventitial tissue.	Normal arterial segments may be present between the arteritis-affected sections of the artery.
Periadventitial inflammation	Aggregates of mature lymphocytes (≥15) are localized around small vessels in the periadventitial connective tissue with no inflammation of the temporal artery.	This pattern may be the only histological evidence of inflammation in a small subset of patients with GCA (<9%). Male sex, absence of halo sign in ultrasound, and PMR symptoms are more likely to occur in patients presenting this pattern. Cranial ischemic manifestations, including blindness, may occur.
Adventitial inflammation	Inflammation in the vasa vasorum and/or inflammation extended to the adventitia without detectible inflammatory infliltrate crossing the external elastic lamina into the media.	May appear as isolated adventitial inflammation in early/mild stages of the disease. Frequently coexists with periadventitial and transmural inflammation.
Transmural inflammation	Concentric rings consisting of mature lymphocytes and macrophages, with a thicker ring in proximity to the external elastic lamina and a thinner ring adjacent to internal elastic lamina. The bulk of inflammation is localized at the adventitia media border. The rings extend from the adventitia to the intima (or the intima-media junction). The adjacent media is relatively spared except in severe cases. Giant cells are usually seen along the internal elastic lamina. Some cases involve laminar necrosis consisting of acellular eosinophilic material along the internal elastic lamina, surrounded by histocytes, whereas fibrinoid necrosis is rare in GCA.	With panarteritis, this pattern is the most frequently seen in TABs. The absence of giant cells does not preclude GCA diagnosis.
Panarteritis	Inflammatory infliltrate in all three arterial layers. The severity and the extent of inflammation is greater than in transmural inflammation.	Indicates severe inflammation. Jaw claudication and scalp tenderness are more likely to occur in patients exhibiting this pattern.
Healed arteritis	Irregular intimal thickening, intimal and medial fibrosis, focal areas of persistent chronic inflammation, multifocal to complete loss of elastic lamina, medial neovascularization, and adventitial fibrosis.	Cautious interpretation of this pattern is required since, as well as indicating healed arteritis, it may be a consequence of normal aging and arteriosclerosis/atherosclerosis.
Atherosclerosis	Regular intimal proliferation, focal loss of internal elastic lamina, calcification of the media (Monkeberg’s calcifications). Absence of significant medial pathology.	These findings may be also be present between arteritis affected sections, and in healed arteritis.

Different patterns may coexist in the same temporal artery specimen.

Small vessel vasculitis (SVV) of capillaries in the connective tissue that surrounds an uninflamed temporal artery is an infrequently reported, but probably underestimated, histological pattern. Every TAB includes a portion of the connective tissue surrounding the biopsied artery that may contain capillaries, arterioles, small nerves and, occasionally, small veins (53). The pattern suggestive of vasculitis consists of an aggregate of mononuclear inflammatory cells (≥15) without polynuclear neutrophils and eosinophils and without the presence of fibrinoid necrosis, surrounding a capillary 0.5–1.5 mm from the arterial wall of an inflammation-spared temporal artery. The small nerves may also be affected (53).

The prevalence of SVV and its clinical significance was investigated in a multicentre prospective study of a cohort of 397 patients with GCA (280 biopsy-confirmed) and 101 patients with isolated PMR (53). Isolated SVV was present in 35 (7%) of 498 patients with clinical GCA or PMR diagnosis. Patients with SVV were more often male and showed fewer systemic and cranial ischemic symptoms and lower inflammatory response compared with patients with biopsy-confirmed GCA (53). Symptoms of PMR were also more frequently observed in patients with SVV in this study as well as in a small retrospective observational study including 28 patients with SVV (54), whereas PMR symptomatology was equally distributed among histological patterns as shown by Cavazza et al. (32) Blindness occurred in one of the 35 patients with SVV (53). Of note, SVV was reported in only 3/35 cases (9%) of the initial pathology reports (53).

Although the histological features of GCA are more varied than previously thought, the finding of SVV should be interpreted with caution, as SVV surrounding an uninflamed temporal artery can also be seen in other vasculitides and malignant disease (57). Cavazza et al. found three of 32 patients to exhibit isolated SVV positive for ANCA-associated vasculitis and one with amyloidosis (32). Thus, when TAB features atypical of GCA histology, such as fibrinoid necrosis or leukocytoclasia, are present, alternative diagnoses may be considered based on clinical, laboratory, and imaging findings (58). On the other hand, the presence of PMR or of other clinical features typical of GCA favours the diagnosis of GCA.

### Healed arteritis vs. atherosclerosis

Caution is advised in interpretation of the TAB when histological evidence of active ongoing arteritis is absent, and the primary findings include healed (quiescent) arteritis. This pattern of histological findings may also be present in atherosclerosis and in normal temporal arteries because of aging. This topic is an area of debate among pathologists (32, 55, 56). It seems that scarring and neovascularization affecting media and adventitia in a temporal artery with no detectable inflammation suggests healed arteritis, whereas isolated effects on intima and internal elastic lamina (Table 2) indicate atherosclerotic or age-related changes (55, 56). A retrospective observational study from the USA examined 400 TABs to investigate the clinical course of healed arteritis (55). Forty-seven biopsies (11.8%) were identified as healed arteritis in the initial pathology report. When published criteria of healed arteritis were applied, only 15 of the 47 cases were confirmed to be healed arteritis (55, 59). Thirty of 47 were categorized as normal or age related/atherosclerotic changes and two as active arteritis (55). Maleszewski et al. in their study of repeat temporal biopsies in patients with GCA under GC treatment observed active arteritic lesions in 60%. Even among those biopsied 9 and 12 months after the initial biopsy, 44% were positive, mostly patients symptomatic at the time of the second biopsy. Thus, histological evidence of active arteritis may be present weeks or even months after the initiation of therapy with GCs and therefore, TAB reports describing findings consistent with healed arteritis a few weeks or months after the initiation of treatment should be interpreted cautiously, as these findings may be primarily related to age-related changes and/or atherosclerosis (45–48, 51, 55, 56, 60).

## Conclusions and future perspectives

Although useful alternatives have emerged, TAB remains the gold standard for cranial GCA. It is a well-established outpatient procedure with very low rates of complications. Modifiable factors as the specimen length and the number of examined sections could increase the sensitivity of the procedure as the specificity is already high. Recent insights in the disease’s pathophysiology, which have elucidated the course of the inflammatory infiltrate within the artery with a clear direction from the vasa vasorum in adventitia to intima, may increase the diagnostic yield of TAB by identifying early stages of the disease with isolated affection of adventitia and/or small vessel vasculitis of the capillaries in the connective tissue surrounding the temporal artery (32, 46, 53). Looking for traces of previous inflammation in the adventitia and in media may be helpful to distinguish a healed arteritis from age-related changes (51, 55). In rare cases, TAB could be helpful tool to identify other diseases which can be presented with cranial symptoms and features of systemic inflammation mimicking GCA such as ANCA-associated vasculitis and amyloidosis (32).

TABs have, together with large vessel imaging, been used to identify patients with definite GCA for clinical trials with bDMARDs, e.g., tocilizumab (61). Additional novel therapies are currently investigated in phase III studies (62). The findings in the TAB may be used in future studies to predict response rates to specific treatments, based on improved understanding of the underlying mechanisms.
